# Reviews

**Published:** 1887-04

**Authors:** 


### REVIEWS.

The Comparative Anatomy of the Domesticated Animals, by A. Chauveau, Professor at the Lyons Veterinary School. Second edition revised and enlarged, translated and edited by George Fleming, F.R.G.S., M.A.I., Veterinary Surgeon Royal Engineers. With 450 Illustrations. Price, $6.00. New York: D. Appleton & Co. 1886.

Professor Chauveau has placed both the medical and veterinary professions under deep obligations to his industrious pen, and has made a valuable contribution to comparative anatomy by giving to the world his full and elaborate treatise on this subject. The medical man, no less than the veterinarian, stands in pressing need of the knowledge which this volume supplies. Function and structure are so intimately related that no one can hope to obtain a knowledge of the former without a thorough understanding of the exceedingly varying character of the latter. Function is stable and unvarying, except in accidental details, while the anatomical structure of which it is the expression presents wide discrepancies and radical differences. The consequence is that unless we contemplate function in connection with the multitude of anatomical differences which present themselves in the animal kingdom, we fail to obtain a full comprehension of its significance and far-reaching effects. It has long stood as a reproach against physicians that their one-sided conception of function, gleaned exclusively from human sources, prevented them from fully understanding the physiological effects of remedies and the wide operation of pathological causes. It is only when function is viewed as the outcome of variable anatomical conditions that its philosophy can be comprehended. Digestion and circulation are perfect physiological processes in in the horse or the cow as they are in man, and no one can be said to understand these processes fully whose knowledge of them is confined to their occurrence in the human subject. Apparent aberrations from a normal condition of these functions are apt to bewilder the physician who knows nothing of comparative anatomy and who has no idea of analogies.

The science of comparative anatomy is our only safe guide in the study of evolution in animals and it is the source of that philosophy which exhibits to us the entire animal world as pervaded by a plan which holds it all riveted together and imparts to it an aspect of profound significance. It was the light which this science supplied that enabled Darwin to elaborate his wonderful scheme of the origin of species, and likewise enabled St. George Mivart to point out the weaknesses of that ingenious theory. In fact physiology, especially that of the nervous system, cannot now rank as a study unless it embrace in its wide grasp the facts of all animal life. Huxley, Bastian, Romanes and Luys could never have executed their grand works had they not built upon the solid ground work of comparative anatomy. The scientific world is therefore justly indebted to Professor Chauveau for his valuable contribution to this branch of study and no less perhaps to Dr. Fleming for his faithful and accurate translation. Many a student of anatomy who recalls the interest with which he listened to the explanation of rudimentary and analagous organisms, and who also recalls the scantiness of the information that reached him, cannot but feel delighted that he here finds ample means of elucidating the meaning of those wonderful co-related structures which simplify as they tend to unify the plan on which the living universe was projected. To sieze the underlying features that bind together the most widely separated species, to perceive the structural unity that pervades the organization of living beings and to trace out the dissimilarities which mark their divergence, is a work which offers the fullest scope to the efforts of the philosophical physiologist. The illustrious Cuvier and the no less distinguished Geoffroy St. Hilaire may be regarded as the originators of this interesting line of investigation, and their labors have lent a wonderful impulse to comparative physiology and anatomy. It was to the futherance of the work inaugurated by those brilliant savants that Professor Chauveau has addressed himself, and if the former laid the foundations of the science deep and broad, the latter has suitably crowned the edifice. Veterinarians can no longer plead that the anatomical side of their profession is incomplete, for Professor Chauveau's treatise has covered the entire ground of veterinary anatomy and leaves nothing for the most ardent investigator to desire. It is to be hoped that the students of both medical and veterinary colleges will appreciate the value of his labors and that his work will become a standard volume in their hands.

C. M. O'L.

---

An Introduction to General Pathology. Founded on Three Lectures Delivered at the Royal College of Surgeons, London, 1886. By John Bland Sutton, F.R.C.S. Price, $4.50. P. Blackiston, Son & Co., Philadelphia. 1886.

Mr. Sutton's aim in these lectures is to apply certain general truths of biology to certain classes of the facts of pathology. Three principles have been propounded by Professor Huxley, as follows: 1, There has been an excess of development of some parts in relation to others; 2, Certain parts have undergone complete or partial suppression; 3, Certain parts which were originally distinct have coalesced.

In illustration of these, the author presents from comparative pathology a mass of examples of original observation, many of them remarkable and *bizarre.* "Excess of development" and hypertrophy are illustrated by hypertrophy of one kidney and atrophy of the other in birds; by the overgrowth of horns, nails and teeth. Atrophy, so frequently correlated with hypertrophy, is expounded by cases testifying to much excursiveness of research; illustrations are taken from embryology, zoology and human anatomy. An interesting example of atrophy in the embryo, observed by Professor Parker in the green turtle, is given. "Parker found that in the embryo of his third stage, about six and a half lines long, fifty-one somatomes exist, but in the adult forty-one vertebræ only are found. The number of somatomes in the dorsal, lumber and sacral regions is equal to the number of vertebræ in the adult, but in the cervical region seven somatomes abort, and in the caudal region three. This singular suppression of parts in the embryo suggests an ancestry with longer necks and tails than are found in the forms which now exist."

Some extraordinary facts are adduced, showing correlated developmental excess from atrophy of the ovaries. When in females the offspring-bearing period of life has passed there is a dwindling of the organs of generation and correlatively an assumption of masculinity. The growth of hair on the face in old women is familiar to everyone, but the following examples are novel and wonderful. Youatt "had an opportunity of examining seven hen pheasants in plumage more or less resembling the male, and in all of which the sexual organs were diseased, but with some variation as to extent. The ovary was contracted in size, of a purple color, and hard to the touch. The oviduct was diseased throughout its whole length." The following case of a female golden pheasant was observed by the author. "A pair of birds, male and female, were given to the society by Sir H. W. Tyler, M. P., but the hen bird presented all the resplendent dress of the male bird, even to a slight yellow coloration on the legs. The ultimate fate of the bird was rather tragic, for in the spring of 1885 she would not yield to the solicitations of the male, and he killed her. The post-mortem examination showed the ovary dwindled to the dimensions of a pea."

Chapter IV is on the correlation of organs. Mr. Sutton espouses the doctrine that division into sexes in animals has been brought about by relatively excessive function of one or the other set of procreative organs in hermaphrodite individuals, hermaphrodism being regarded as the primitive sexual condition in the evolutional history of animals. The exclusive use of one set having led to the other becoming obsolete. This is a beautiful example of the working of the principle of the correlation of hypertrophy and atrophy.

The author defines inflammation as "the method by which an organism attempts to render inert noxious elements introduced from without or arising from within." In dwelling at considerable length on the power possessed by the leucocytes of inflammation to digest and dispose of noxæ, mention is called to the recent work of Metschnikoff on intracellular digestion. The story of inflammation is "likened to a battle.

The leucocytes are the defending army, their roads and lines of communications the blood-vessels. Every compositic organism maintains a certain proportion of leucocytes as representing its standing army. When the body is invaded by bacilli, bacteria, micrococci, chemical or other irritants . . . . . the leucocytes rush to the attack. In the conflict cells die and often are eaten by their companions ; frequently the slaughter is so great that the tissue becomes burdened by the dead bodies of the soldiers in the form of pus."

If we may be permitted a gentle criticism here, this does not, it appears to us, set forth any new conception, in the scientific sense, of the process of inflammation. It is a purely human though interesting way of looking at the facts ; it does not as a scientific concept should "take and lay hold of on all sides" all the essential phenomena. How large an anthropomorphic element there is in the notion is seen in its want of correspondence with nature. The cause of inflammation may be something which it is impossible for leucocytes to devour, as the electric current, or thermal vibrations. If it be said that the function of leucocytes is to eat up their own dead, it may be replied that death of tissue elements is not essential to inflammation. The necessary lesion is in the vessels ; other phenomena, as the exudation of leucocytes, being largely secondary. Also the process of recovery or resolution may, in slight inflammations at least, take place quite independently of the action of leucocytes. It is scarcely legitimate to speak of leucocytes as acting with conscious, or instinctive, or reflex intelligence ; these are the attributes of nerve tissue, though, doubtless, they are forshadowed in the seemingly intelligent actions of simple protoplasm. A rational and philosophical explanation of inflammation must be made in the terms of physics.

We have not space for a detailed notice of remaining chapters. Cystomata and neoplasms are considered with a remarkable fullness of new knowledge and many dark places in the pathology of new formations are brightly illuminated. The author has rendered a very important service to pathology by publishing this record of his exceptional experience. Since 1878 he has systematically examined 12,000 animals from fish up to man.

C. P. M.

Dell Identita della tisi perlacea dei bovini colla tuberculosi Umana e della sua Contagiosita. G. Nuvoletti, Drucker and Tedeschi, Verona and Padova, 1887.

This recent Italian contribution to the literature of tubercular diseases is well timed and interesting. It is not many years ago since public opinion was in a state of complete lethargy respecting the transmissible character of these diseases, and, though some earnest spirits contended that it was a question intimately connected with public health, the matter remained practically in abeyance and no steps were taken to protect the human species or the lower animals from the danger of contagion.

The advances made in microscopy, however, together with the astonishing strides taken in bacteriology, have drawn earnest attention to the contagious character of tuberculosis and to its communicability from one species to another. The rapid and alarming spread of tubercular maladies has indeed filled men with apprehension, especially as all recorded attempts to put a stop to its ravages had proved completely ineffectual. So appalling had become the severity of this scourge, especially in large cities and crowded centres of population, that its victims were counted by the hundreds of thousands, and epidemics paled into insignificance beside its destructiveness. Ringel has said: "It is impossible to foresee what will be the condition of humanity in a very short time unless some effective measures be adopted to stem the inroads of this tide which threatens to engulph the entire human family."

The treatise, title of which heads this notice, addresses itself intelligently to the consideration of these two questions: 1st. Is the peculiar phthisis of horned cattle identical with human tuberculosis?

2d. Is this same phthisis communicable from one individual to another who use the flesh and milk of tubercular cattle. To both these questions an affirmative reply is given and abundant data have been gathered from all sides to constitute an ample and convincing proof.

As far back as 1865 Professor Villemin in a communication sent to the Paris Academy of Medicine proved that the inoculation of tubercular matter resulted in the transmission of the disease from one zoological species to another and that the same order of phenomena was observed as attended the transmission and development of all virulent maladies. Experiments similiar to those of Villemin were conducted from time to time by Herard and Cornil in France, by Perroncito in Turin and by Pavaskera and Tallonis in Greece. The experiments of Chauveau, however, were the most complete and satisfactory, so that no doubt longer lurks in the minds of the most skeptically inclined touching the ready transmissibility of tubercular diseases by innoculation. Experiments to the same effect were made and continued by Haselbach, Scholz, Klebs and Colin from which the following valuable conclusions have been deduced:

1st. Bovine tuberculosis is absolutely infectious.

2d. Tubercular matter taken from the other organs of the body do not possess the same degree of infectiousness as that taken from the lungs.

3d. Infection can take place both by inoculation and by the ingestion of infected material.

4th. There can be no doubt as to the identity of bovine phthisis with the human tuberculosis.

5th. That the flesh of animals affected with tuberculosis possesses, though in a less degree, the same infectious qualities as tubercular matter itself.

6th. That boiling destroys the infectious principle; but that even after boiling tubercles continue to possess a certain amount of virulence, just as in the case of trichinosis.

7th. That the infectious character of the milk of tubercular cattle has been incontestably established.

These conclusions possess a wonderful significance and should be seriously taken to heart by sanitary veterinarians.

It is evident that the stamping out process cannot be too rigorously enforced; that suspected cattle should be immediately slaughtered, scrupulous cleanliness observed and such hygienic measures put in operation as are calculated to keep the health of cattle *in transitu* up to the highest standard; considerations of general economy and human health demand nothing less. C. M. O'L.

